# The apple of discord: can spider cocoons be equipped with antimicrobial factors?—a systematic review

**DOI:** 10.1186/s12983-025-00563-5

**Published:** 2025-05-28

**Authors:** Mateusz Glenszczyk, Artur Lis, Weronika Porc, Magdalena Pacwa-Płociniczak, Agnieszka Babczyńska

**Affiliations:** 1 Environmental Stress Biology Team, Institute of Biology, Biotechnology and Environmental Protection, University of Silesia in Katowice, Bankowa 9, 40-007 Katowice, Poland; 2 Environmental Microbiology and Bioremediation Team, Institute of Biology, Biotechnology and Environmental Protection, University of Silesia in Katowice, Jagiellońska 28, 40-032 Katowice, Poland

**Keywords:** Spider cocoons, Egg sacs, Antimicrobial agents, Antimicrobial properties, Environmental microbiology, Interspecific interactions, Evolutionary biology, Coevolutionary dynamics

## Abstract

The antimicrobial properties of spider silk have been a topic of scientific intrigue since ancient times. Despite extensive research, the question remains unresolved due to conflicting findings and methodological challenges. This work revisits and synthesizes current knowledge, proposing that spider cocoons, rather than other spider products, serve as a particularly promising focus for investigating antimicrobial factors. This emphasis arises from their critical role in parental investment and reproductive success, as the maternal care associated with spider egg sacs suggests the necessity for enhanced antimicrobial protection to safeguard offspring. By investigating existing research, we propose that the protective properties of spider egg sacs may derive not only from the silk itself, but also from the eggs contained within, as supported by previous hypotheses. Furthermore, drawing on the body of knowledge, we suggest that potential antimicrobial defense mechanisms may extend beyond intrinsic factors, encompassing interactions with microorganisms, plants, and other possible environmental elements that remain unexplored but may likely be interconnected. This review highlights that the potential interplay of these factors may be complex and possibly influenced by ecological and biological contexts. Unraveling these dynamics requires an interdisciplinary approach, incorporating diverse methodologies and perspectives to address the gaps in current knowledge. By refining the focus and embracing a broader conceptual framework, future research can provide definitive insights into the antimicrobial properties of spider cocoons. Resolving this long-standing question will not only clarify the scientific debate but also deepen our understanding of spider biology and the adaptive strategies that have evolved to ensure reproductive success.

## Introduction

Since the ancient times antimicrobial properties have been attributed to spider silk. Spider silk was supposed *materia medica animalis* (a medicine of animal origin) and was used in a number of treatments. Depending on the assumed therapy an external or internal supply was envisaged. For external treatment purposes spider silk was used to address i.a., dermatologic, ocular or rheumatological conditions, while internally it was used to i.a., stop hemorrhages, treat asthma or as a tooth filling. In addition, the spider web was used as an ingredient in the preparation of medicinal concoctions or as a patch to hold the administered medicine in place. It was also believed that spider web coming only from particular tree species have treatment capabilities [22, 46, 113, 137]. It was unknown whether spider silk actually had curing potential or it was a mere placebo, so an effect one wanted to believe in. In 1995 a term called “Arachnicillin” emerged [44] followed by a several articles and forums using this word in the upcoming years, showing that dilemma around spider silk continues to persist in contemporary times and has not been fully resolved.

Throughout the years, this area of research has become an apple of discord, as various studies yield differing, often contradicting conclusions. Most of the reports are fairly recent [5, 15, 92], some [61] present inconsistent results, others [100] require methodological clarity due to the usage of solvents without considering their antimicrobial activity [37]—Table 2), or exhibit lack of control groups for contamination [6, 164, 165]. Some and other of these contradictions and methodological limitations have been summed up and evaluated by another research group studying spider silk [40]—Table 1). Their findings challenge the hypothesis that spider silk possesses antimicrobial properties, though even then, the authors do not fully exclude this possibility, suggesting that other, as yet untested spider species may exhibit such properties or that spider silk could be effective against microbes not examined in the studies. It is important to note, that by identifying and synthesizing key methodological challenges, Fruergaard et al. have significantly advanced this area of research, underscoring the need for further methodological refinement and exploration across diverse species and microbial targets. The last issue is especially important. Careful consideration of the specific types of microorganisms used in experimental design is essential in this area of research. Microorganisms include both prokaryotic and eukaryotic forms, yet to date, most studies have primarily focused on prokaryotic microorganisms, particularly Gram-positive and Gram-negative bacteria. Even within a single bacterial species, strain-level differences can lead to significantly different outcomes. The use of standardized microbiological reference numbers (e.g., National Collection of Type Cultures—NCTC) should represent the best practice—especially in antimicrobial susceptibility testing—yet it was often omitted. Beyond prokaryotes, there are also eukaryotic microorganisms, such as fungi, which have received comparatively less attention, despite their likely significance, as even greater environmental challengers for developing spider embryos within the cocoon.

Research has demonstrated that spiders produce various types of silk, each derived from distinct glands specialized for specific functions. Notably, cocoon silk, which is used to enclose and protect a cluster of eggs, results from the combined secretions of two specialized gland types. This silk plays a crucial role in safeguarding spider embryos, which are rich in energy-dense compounds that can serve as a substrate for potential pathogenic bacteria and fungi. Given their protective function, spider cocoons—often referred to as egg sacs or egg cases—represent a promising material for further studies [15, 39, 40]. There is still little known about the diverse types of cocoons among spiders. Numerous species build different types of cocoons, in various habitats, each exhibiting diverging macro- and microscopic characteristics in their silk thread [158]. It is currently unclear whether cocoons serve not only as physical barriers due to their dense silk structure and hydrophobic properties [15, 92, 159], but also as biological barriers through the potential presence of antimicrobial agents.

If antimicrobial agents are indeed present in spider cocoons, do they localize exclusively on the silk threads, or are they also found on the energy-rich eggs within the cocoon? Moreover, do they exhibit variability in their distribution or efficacy (e.g., different on the cocoon’s silk and different on the cocoon’s eggs)? Furthermore, is there a volatilome (i.e., a compound cloud) encompassing the cocoon silk, akin to the phenomenon described by Lammers et al. [77], where the scientists documented the existence of an antimicrobial volatile cloud around communal silk in social spider species. If so, could this be linked to the cocoon’s potential microbiome? Finally, could the habitat and life history of spider species significantly influence their antimicrobial potential? At present, there appear to be no studies providing a comprehensive view on this matter, current understanding is primarily derived from fragmented findings. The broad scope of the question posed in the title necessitates a multi-layered analysis, which we are motivated to provide. We acknowledge, however, that additional dimensions of this topic may emerge beyond those explicitly addressed. Nonetheless, we think that advancing research in this area remains imperative, particularly given the growing challenge posed by the phenomenon of antibiotic resistance.

The global explosion of broadly antibiotic-resistant bacterial pathogens is a relatively recent event [7, 122]. It occurred as a side effect of several overlapping factors from which, the most significant are: (i) the widespread production of antibiotics, (ii) extensive use of antibiotics across various domains of human activity, including clinical medicine, veterinary medicine, agriculture, aquaculture, horticulture, (iii) microbial adaptability, including horizontal gene transfer [7]. This issue is worsened by a lack of new drug development by pharmaceutical companies and research institutions, a situation driven by diminished financial incentives and stringent regulatory requirements. There is a pressing need for coordinated efforts to establish new policies, reinvigorate research, and take measures to manage the antimicrobial resistance crisis [156]. Furthermore, an increasing body of research suggests a correlation between the rate of emergence of antibiotic resistance and the rise in global temperatures [26, 85, 94], which suggests that the crisis will be further amplified by another set of emerging and progressing factors regarding climate change. In addition, the term “silent pandemic” has gained attention in the context of the SARS-CoV-2 virus, referring to a quick global increase in pathogens’ resistance to antibiotics due to irresponsible antibiotic prescriptions for viral infections and improper usage during worldwide lockdown [79, 90, 98]. Discussion about this problem seems to extend beyond multidrug resistance (MDR and encompass extensively drug-resistant (XDR and pan-drug resistant (PDR microorganisms (Magiorakos et al. 2012 [118]).

In response to the current drug resistance crisis, exploring new sources of antibiotics has become a pressing necessity. Among the underexplored sources, animal-derived compounds have attracted particular interest due to their unique bioactive properties. Within this realm, special attention is being given to fatty acids (FAs), and antimicrobial peptides (AMPs). Apart from that, animal microbiomes (AMBs) have recently emerged as another critical focus of research due to their pivotal roles in shaping immune system functionality and bolstering microbial defense mechanisms in animals [16, 111, 125, 153, 161].

FAs are a class of lipids that have been known for their antimicrobial properties since the late 1880s [145, 169], which, due to the discovery and the development of traditional antibiotic drugs, were neglected for a long time [161]. Among others within this group caprylic acid (8:0), capric acid (10:0), lauric acid (12:0), myristic acid (14:0), palmitoleic acid (16:1), oleic acid (18:1) were documented to effectively cause the reduction of colony and inclusion-forming units in vitro [145].

FAs possess the ability to penetrate cell walls and disrupt the integrity of cell membranes, leading to the release of membrane-associated proteins. This disruption interferes with electron transport chains, uncouples oxidative phosphorylation, and inhibits key enzymatic activities, collectively resulting in impaired nutrient uptake and respiratory function, ultimately inhibiting microbial growth [29, 76]. Additionally, FAs have been shown to downregulate genes associated with biofilm formation [161]. It has also been observed that microbial cells in the logarithmic growth phase are particularly sensitive to FAs compared to cells in other growth phases, which only supports their role in reducing microbial cell numbers [145]. FAs at lower concentrations exhibit a primarily reversible bacteriostatic effect, however, at higher concentrations, their detergent-like action exerts an irreversible bactericidal effect [76]. Moreover, FAs are said not to exist in a free state, due to their high affinity to a number of proteins [49] and synergistic effects with other compounds like antimicrobials [23, 120, 140, 169] or emulsifying agents, which further increase their antimicrobial potential [145]. Some of the previously mentioned examples of fatty acids were discovered both in hunting (i.a., capric, lauric, myristic, palmitic, stearic) [171] and cocoon silk (i.a., stearic, palmitic, oleic) [132]. In the latter case, the authors pointed out that these agents tended to dissipate in the later post-embryonic period [132]. This observation raises important questions regarding the functional role of FAs during the early stages of embryonic development, as well as the temporal dynamics of cocoon silk composition and its potential adaptive benefits in offspring protection.

Antimicrobial peptides (AMPs) are low molecular weight proteins that play a critical role in host innate immunity, providing defense against a broad spectrum of microorganisms [170]. AMPs exhibit two primary mechanisms of action: membrane-mediated and membrane-independent. In the membrane-mediated mechanism, AMPs disrupt membrane permeability, compromising cell integrity. In the membrane-independent mechanism, AMPs translocate across the membrane to target intracellular processes. There, they can inhibit the synthesis of nucleic acid, proteins, alter enzymatic activity, or interfere with proteolytic processes. They can activate autolysins, phospholipases, and inhibit septum and cell wall formation by interfering with peptidoglycan synthesis [3]. The wide influence of AMPs on essential cellular processes cumulatively leads to cytotoxic effects and either microbial inhibition or death. In addition to their antimicrobial effects, AMPs exhibit a broad spectrum of activities, including immune regulation, angiogenesis, wound healing, and antitumor properties [3, 138, 170]. A comprehensive collection of AMPs can be explored in the Antimicrobial Peptide Database (APD) (https://aps.unmc.edu/home), which, as of November 2024, includes 42 spider-derived AMPs. While the majority of entries focus on peptides derived from venom glands, a preliminary study by Molenda et al. [103] documented the presence of AMPs, including lysozymes and defensins, in the cocoon eggs of *Parasteatoda* and *Pardosa* spiders. Notably, in their studies, protein levels increased progressively with embryonic development, suggesting a temporal dynamic in the cocoon’s adaptive protective mechanisms, akin to those observed for fatty acids. Additionally, the authors mentioned other low-molecular-weight proteins within the cocoon, which may contribute further to its defense.

Microorganisms can inhabit animal surfaces, whether external or internal, forming intricate and dynamic ecosystems. Given the metabolic richness of microorganisms and their diverse biosynthetic pathways, AMBs may represent a prolific and largely unmined source of not only novel antibiotics but also other bioactive compounds, which may alter the growth of microorganisms [2]. Despite their ubiquity, the nature and specificity of many microorganism-animal host associations remain poorly characterized, underscoring the need for further investigations. In this context, the most extensively studied systems seem to be those of the human gut, which have also led to the discovery of new antibiotic compounds [43, 148]. In spiders, however, comprehensive metabolic profiling of microbial communities remains limited, leaving their functional potential largely uncharted. Theoretically, such relationships involve the animal host supplying the microbial community with nutrients and maintaining relatively stable environmental conditions, while the microorganisms, in return, provide the host with enhanced protection against pathogen invasions [16]. In the context of spiders’ egg sacs, an intriguing question is whether land-dwelling spiders (e.g., *Pardosa*) recruit soil microorganisms to their egg sacs to harness their antibiotic properties, potentially bolstering offspring protection while simultaneously offering these microorganisms a nutrient-rich, stable environment within the cocoon. Another critical area of inquiry is the potential existence of obligate symbionts—microorganisms strictly associated with the cocoon that are incapable of surviving outside of it. Furthermore, the role of ecological niches and climatic factors in shaping the antimicrobial potential of spider egg sacs remains poorly understood, as do the mechanisms that might drive interspecies variations. Currently, there is a lack of detailed studies addressing these questions in the context of spider cocoons. Investigating the microbial communities associated with egg sacs offers an opportunity to unravel the intricate ecological relationships between microorganisms and their hosts. It is important to note that studying these interactions may be complicated due to the presence of both cultivable and uncultivable microorganisms within the cocoon. Thus, genetic approaches such as metagenomics or 16S rRNA sequencing could provide a more comprehensive insight into the microbial diversity associated with egg sacs. These methods should allow for detailed exploration of the full range of microorganisms present, including those that cannot be cultured in vitro, and help to clarify the composition and functional roles of microbial communities in these complex host-microbe interactions. Such research could also pave the way for the discovery and characterization of novel bioactive compounds. By identifying the microbial species present and elucidating their roles as either symbionts or pathogens, these studies may provide significant insights into the resistome of spider egg sacs [16, 63, 88, 119].

Building on the outlined considerations, we propose that spiders and their cocoons constitute an excellent model for exploratory research, with many topics remaining unresolved. Whether the ultimate findings of this "apple of discord" confirm or refute the presence or absence of antimicrobial compounds, either outcome stands to significantly deepen our understanding of spider biology. This dual potential underscores the value of such investigations, presenting an opportunity to expand knowledge regardless of the conclusion. As for the following sections, we aim to consolidate existing information and offer a distinct perspective on the topic by incorporating often omitted ecological aspects. Whether spider cocoons can or cannot be equipped with antimicrobial factors is a question requiring taking into account ecological interactions that spider species face with in a coevolutionary arms race. Finally, by addressing some of the complexities in this field, we hope to lay a more profound framework, which will inform and further guide the research in this still unresolved topic.

## Spider egg sacs

### Parental investment and maternal care

Parental investment as defined by Trivers [151], encompasses any allocation of resources or effort by a procreating organism to enhance the survival and reproductive prospects of their offspring, even at costs to the parent [130]. In spiders, this investment predominantly falls on the females rather than males, leading to females exhibiting greater selectivity towards males, which is consistent with the theory of mate choice [25, 38]. Shortly after insemination, females retreat to or organize their hideout, where they start to construct and manage their cocoons [18, 39]. Spider maternal care consists of two components: constructing behavior (CB) and management behavior (MB) occurring consecutively [39, 62, 131]. CB consists of several stages, where females create a basal plate, cylindrical walls, deposit eggs inside, and coat the egg sac chamber with a cover plate. Following this initial assembly, a series of finishing steps occur, which include adding additional loops of threads to the top of the cover plate or creating suspension threads to attach the cocoon to the nearby elements of the environment (e.g., leaves, twigs). Occasionally, an intricate network of camouflage may be interwoven, further enhancing the cocoon’s adaptive properties within its surroundings [39]. Interestingly, the camouflaging pigments were hypothesized by authors of another study to play a protective role against external microorganisms [17, 32, 131]. We find this is especially interesting, as in *Pardosa* genus (which contains 534 species), there are species that seem to not produce cocoon pigments at all (e.g., *Pardosa bifasciata*), and in such cases the egg sac remains white/ivory in color, while typical egg sac of other species in this genus varies from yellowish brown to dark bluish. It is believed that females are able to perceive and judge the condition of their cocoons, which results with adding repairs to severed threads or even eating the egg sac if it is abnormal [65, 66], which could probably function as a way of cessation of further parental expenditure and regaining part of the already allocated resources. In addition, certain spider species (e.g., *Cyrtophora citricola, Cupiennius salei*) seem to demonstrate a higher degree of adaptability of CB, exhibiting the capacity to modify their activities in response to changes in environmental conditions, which may be exhibited by resuming the construction after a pause or adjusting position accordingly to the alterations of cocoon’s angle [70, 97]. Conversely, other species appear less adaptable, and when confronted with such changes, they tend to start the CB all over focusing on a new cocoon [39]. After completion of cocoon construction, MB occurs, during which female spiders typically nurture and protect the cocoon until the emergence of juvenile spiders. Notably, in certain genera (e.g., *Pardosa*) females travel with cocoons and perforate them to allow the offspring to emerge [131]. Furthermore, MB may extend beyond cocoon protection, when females continue to provide resources to their offspring after their emergence and up until their dispersal. These strategies are usually very costly to the female [17, 39, 62, 168]. In extreme cases, a matriphagy is observed, where the female is consumed by her offspring in order to provide a massive amount of nutrition to the juveniles [68, 149].

### Eggs and their protection.

Eggs are provided with rich nutritional resources of organic compounds, including proteins, lipids, carbohydrates, vitamins, and minerals, existing in various proportions. They act as precursors for the formation of lipovitellins, which serve as essential nutrients for developing embryos [71, 73, 74]. The number of eggs inside of spider cocoon differs from genus to genus. For comparison, *Pardosa* spiders lay about 25–45 eggs [150], while *Parasteatoda* can lay up to 400 eggs [54, 95, 101]. Besides, females will lay an additional number of trophic eggs into the egg sacs, which serve as an extra source of nutrients for the juveniles [67].

Embryonic development within spider egg sacs can be broadly divided into two principal stages: the embryonic period (EP) and the post-embryonic period (PEP). In wolf spiders, each stage lasts for up to 15 days [150], which means that the entire cycle from egg deposition to the emergence of juveniles in this spider group takes approximately one month. These stages appear, to some extent, correspond with the previously mentioned CB and MB, with EP paralleling with CB period and PEP with MB. It is important to emphasize that there are significant developmental differences across all spider species. Generally, spiders inhabiting warmer environments exhibit slower development at lower temperatures, whereas those from colder habitats show delayed development at higher temperatures [82]. Moreover, embryos in certain genera (e.g., Linyphiidae) require an additional period of diapause (i.e., developmental dormancy), which further influences the span of whole developmental cycle [53, 136]. Climatic factors, particularly temperature, in relation to species-specific life history traits, ecological strategies, and microhabitat conditions, have been documented to exert a significant impact on the rate of development of spiders [82, 110]. Currently, it remains unclear though, how these factors would influence possible antimicrobial potential, as studies addressing and describing the biochemical and metabolic processes associated with spider development and reproduction are limited [128]. It could be suspected, however, that irrespective of the absolute duration of ontogenesis, the most critical factor for antimicrobial activity could be the actual developmental stage at which the embryo is, as it will dictate the levels and management of energetic and regulatory substrates, including those with immunological role and potential antimicrobial properties [128, 150].

**Fig. 1:**
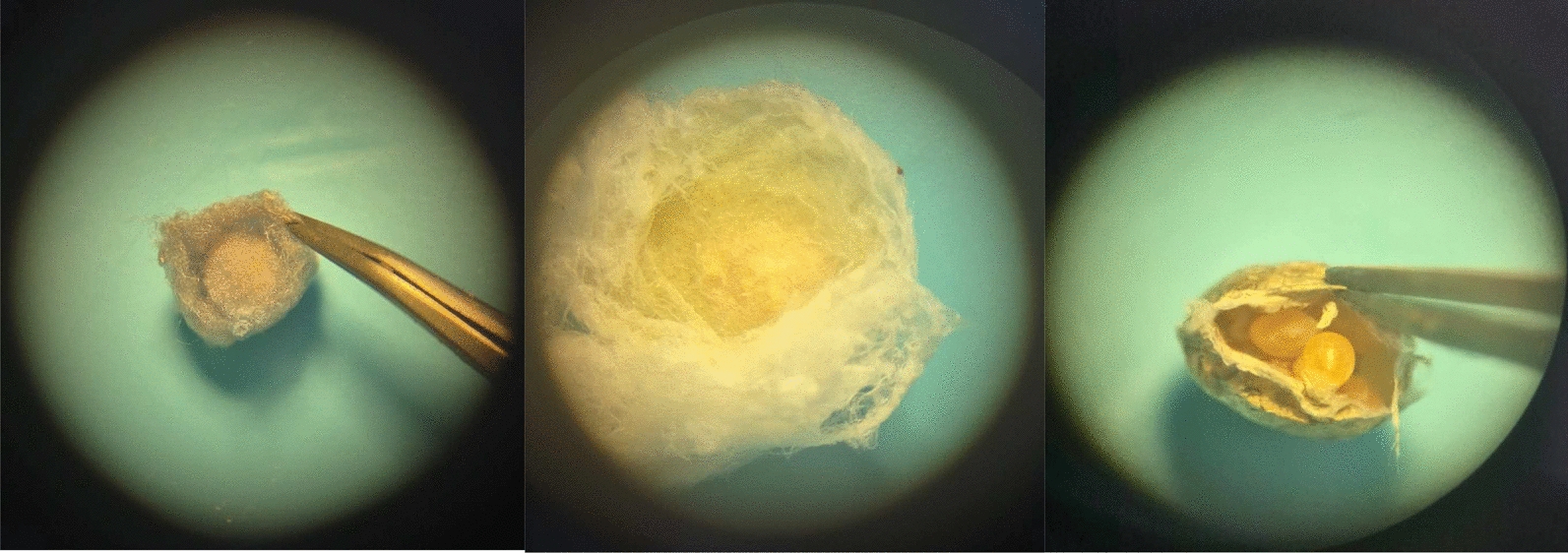
Photographs of opened spider cocoons. *Parasteatoda, Eratigena, Pardosa.* In the first two—cocoon’s silk core present; in the last one—cocoon’s core absent. Photographs by Mateusz Glenszczyk (Author)

In the cocoon the eggs form a clutch, which is held together by a milky-white mucus also called oviposition fluid which disappears within a short time after oviposition, after that the eggs retain softly bonded together by the remaining residues of the mentioned secretion mixed with inner cocoon’s silk threads [31]—Fig. A). We refer to this structure as “cocoon’s silk core”. Based on our observations (Fig. 1), the core is clearly distinguishable in some genera, such as *Eratigena* or *Parasteatoda*, as the inner silk threads that envelop it usually differ in color and other properties from the rest of the cocoon. For example, in *Eratigena* the cocoon’s silk core is created by yellowish, tight-knitted silk threads which differ from the cottony white threads that surround it. In *Parasteatoda* the core is created by grayish cottony threads, which are distinguishable as the rest of the cocoon threads remain fawn. In some genera like *Pardosa*, the cocoon’s silk core is absent, and the eggs are easily accessible after unsealing the pronounced seam running around the cocoon.

**Fig. 2:**
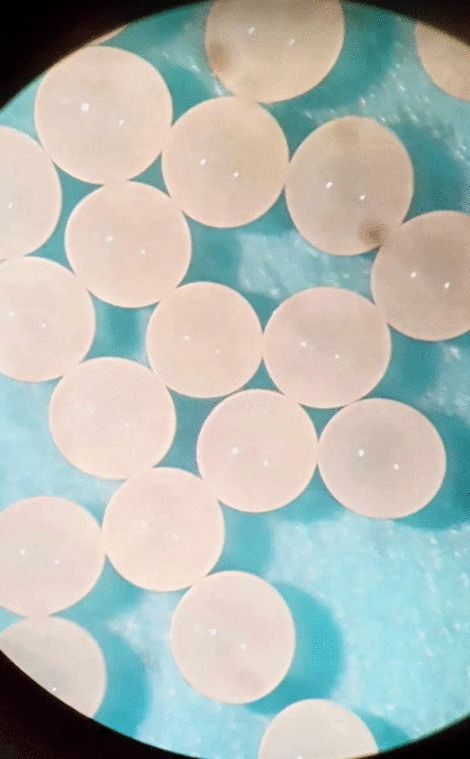
*Pardosa sp.* cocoon’s eggs shortly after oviposition. Photograph by Mateusz Glenszczyk (Author)

Cocoon’s eggs are typically spheroid in shape, and come in various colors depending on species. From orange, through yellow to white (Fig. 2). Typically, they are alabaster/ivory [31]. In addition, we have observed that eggs change their color with time, with alabaster/ivory color shortly after oviposition, and yellowish/orange during later stages of development. Other than that, the eggs were documented to be thin-layered within a relatively stiff parchment-like cocoon [14].

**Fig. 3:**
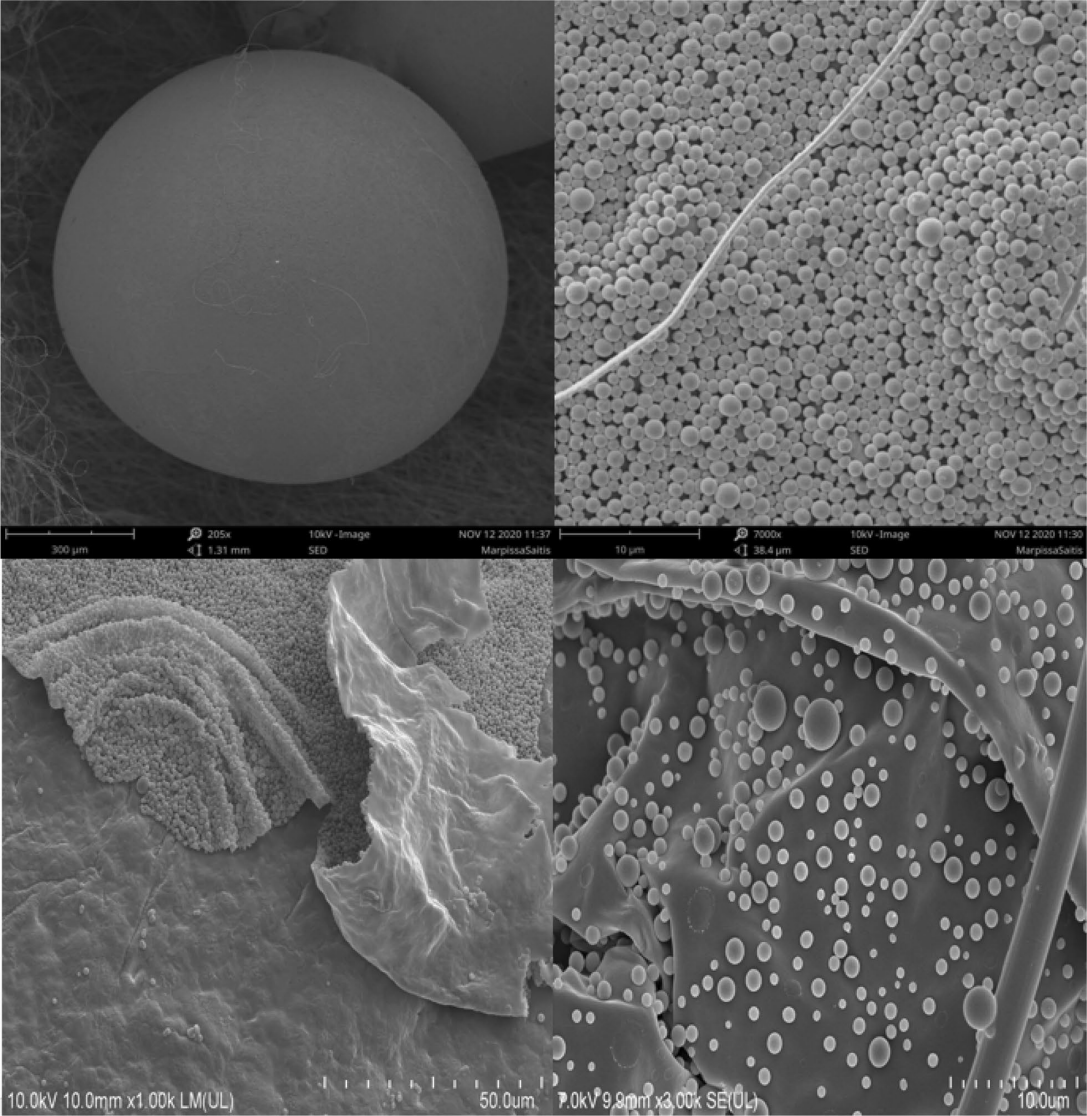
Scanning electron micrographs (SEMs) of spider eggs (material from salticidae, theridiidae): intact egg; surface structure displaying the granular membrane; granular membrane after removal; regions of granular formation on the membrane, together with regions after granular fall off. Top photographs by Mateusz Glenszczyk (Author). Bottom photographs by Izabela Potocka, used with author’s permission

Eggs are coated with a layer of chorion, which is formed in the genital tract. Chorion can be differentiated further into: exochorion, with characteristic granular structures, which come in different sizes and form sparsely or aggregated arrangements without any particular order, and an endochorion, a layer that lies under the exochorion, which covers the vitelline membrane (Fig. 3) [31]. Morishita et al. [108] have documented that the granules of the chorion are produced within the oviduct and are later mixed with lustrous material within the uterus. Contrary to that, Michalik et al. [99] have pointed towards the uteral production of these structures, and Humphreys [60] did not find chorion granules within the spider’s oviducts. Given that, we show a favorable stance toward the notion that the very possible differences in place of production, together with discrepancies in size and density of chorion granules may represent of what is known as a “fingerprint” of species [31, 47, 48, 60]. Granular structures of exochorion are hypothesized to play a protective role towards the egg, similar to what was documented in many insect species [35, 87]. Interestingly, no other arachnid shows similar characteristics, meaning that this phenomenon is exceptional and remains evolutionary conservative to *Araneae* only and is an attribute of some importance [59]. Yet it is essential to note that there is limited data about this matter, exclusively for spiders. Conti et al. [31] suspect that granules of exochorion help to absorb water needed for development of the embryos, while the endochorion beneath prevents dehydration and serves a mechanical function. Makover et al. [92] noted that granules are, in fact, superhydrophilic, while the rest of the egg surface is hydrophobic in nature. In this study it was also documented that spheres bear a positive charge, which should reduce the surface adherence ability of bacteria and consist of low molecular weight proteins which could point towards the existence of potential antimicrobial peptides (AMPs). Humphreys [59] noted that spheres bear the ability to adhere to the parasites emerging from the cocoon eggs. These studies point towards the confirmation of hypotheses that exochorion granules may play a defense role for the eggs.

Eggs of certain spider species have been documented to contain proteins with broad-spectrum antimicrobial activity against both Gram-negative and Gram-positive bacteria. Buffkin et al. [24] investigated the toxicity of black widow spider (*Latrodectus hesperus*) eggs. Their findings indicated that toxicity was present only in the eggs of *Latrodectus* species, whereas eggs from *Loxosceles* and *Araneus* showed no toxic effects. The study also quantified lethal doses (mg/kg) for egg sacs of varying ages. Interestingly, Buffkin et al. referenced [69], who reported the presence of toxic compounds also in the unhatched eggs. Buffkin et al. concluded that the "poison" found in eggs differs from venom. This claim is supported by [4], who demonstrated that extracts from *Latrodectus tredecimguttatus* eggs contained toxins immunologically distinct from venom gland-derived toxins. Yan et al. [167] further elucidated that these egg-derived toxins are proteinaceous, comprising both high-molecular-weight (> 10 kDa) and low-molecular-weight (< 10 kDa) peptides. Li et al. [83] observed that eggs contain more proteins with the catalytic activity and binding functionality than venom, showing distinct, more complex toxicity mechanisms. Subsequent studies by Li et al. [84] led to the isolation of novel toxic proteins from *Latrodectus tredecimguttatus* eggs, designated Latroeggtoxin-I and Latroeggtoxin-II. In later work, Lei et al. [81] expanded this repertoire by isolating Latroeggtoxin-III and Latroeggtoxin-IV, which were evaluated for antimicrobial properties. The latter demonstrated significant inhibitory activity against a range of bacterial pathogens, including *Staphylococcus aureus*, *Salmonella typhimurium*, *Bacillus subtilis*, *Escherichia coli*, and *Pseudomonas aeruginosa*. Antimicrobial assays utilized protein-coated discs alongside ampicillin as a positive control, highlighting the potential of these compounds as broad-spectrum antimicrobial agents. Last but not least, the authors conducted transcriptome and protein database search, and observed that Latroeggtoxin-III shows high homology to that of vitellogenin. The observation of this team shows that in the case of black widow spider, the eggs are equipped with their own individual defense mechanisms.

### Silk

Modern studies have documented silks in spiders are produced by seven different glands depending on their intended usage: aciniform (prey cache cocoons, egg sac internal layer—basal plate); aggregate (sticky coating on a capture frame of web); major ampullate—MA (web outer frame, dragline); minor ampullate—MI (web reinforcements); flagelliform (web capture frame); piriform (web-substrate attachments) and tubuliform also known as cylindriform (egg sac external layer—cover plate) [19, 39, 42, 52, 57]. Research conducted in molecular science have documented that a single silk fibre is made of protein building blocks coming from one family called spidroins [13]. Typical spider silk fibre is made from MA spidroin nanofibrils, which are wrapped up together and coated by a MI spidroin layer (also described as “Skin”), forming an inner covering which is topped by a glycoprotein layer covered by a lipid coating forming an outer covering. This outer covering is thought to function as sericin, which in insects was documented to have antimicrobial properties [127]. It is important to highlight that most of studies researching spider silk are focused mostly on *Aranoidea* spider superfamily, which functions as a model example, but comprises only about quarter of spider species. The amount of data about the diversity of gland morphologies and properties of the silk in other spiders is scarce. Fortunately, recent research slowly changes the state of current knowledge in this area (e.g., [141]). Moreover, there also seems to be a rising awareness in this matter with new projects undertaking to catalog spider silkomes (https://spider-silkome.org/) [9], but as for now, they are not focused around investigating possible antimicrobial properties. Spider silk is thought to be antimicrobial, meaning it is presumed to exhibit both antibacterial and antifungal properties. There are multiple studies, that document this effect or oppose this notion, showing no antimicrobial resistance [5]. This, unfortunately, makes this subject problematic and challenging to navigate through. This topic, together with methodological limitations, was already described in Fruergaard et al. [40], and further reviewed by Schwenck et al. [137]. To avoid unnecessary redundancy, we will refrain from exploring it further here. So far, there seems to be no scientific consensus on this matter. As mentioned before, spider egg sacs are mainly composed of aciniform and cylidriform types of silk. Zortea and Fischer [172] have observed delayed prey decomposition in prey cache cocoons (i.e., cocoons encapsulating victims), which are mainly composed of aciniform type of silk. While it shows rather a microstatic than an antimicrobial effect, this observation opens a question about similar potential in egg sacs, given the fact that they too are composed of this type of silk. It has been proposed that the silk of the cocoon serves to shield the eggs from a range of threats, both abiotic (e.g., temperature fluctuations, humidity) and biotic (predators, parasites) [11, 12, 39]. Babczyńska et al. [15] suggested that it acts as a microbiological sieve preventing potential pathogens from getting through, and Vetter et al. [159] have documented that water-based pesticides cannot effectively penetrate the cocoons due to the hydrophobic nature of thevtightly knit egg sac’s silk. Ruhland et al. [131] discuss the pigments deposited on egg sacs silks by female spiders, hypothesizing that they may play a protective role against microorganisms. As for now, it was documented that silk pigments contain quionones, phenols, porphyrins, carotenoids, and xanthuric acid [21, 41, 55, 56, 123]. Quinones and their derivatives are a large family of natural compounds showing certain colors such as yellow, orange, brown, red, and others. They are documented for a broad range of bioactivity, including antimicrobial capability [36, 135]. The identified mechanisms include irreversible binding with nucleophilic amino acids in proteins, leading to their functional modification or inactivation,disrupting ribosomal machinery by inhibiting S4 protein synthesis; impairing surface-exposed adhesins, cell wall polypeptides, and membrane-bound enzymes; and depriving microorganisms of essential substrates [135]. Moreover, these compounds have been observed to inhibit biofilm formation, in a process believed to rely on the generation of reactive oxygen species (ROS) [34, 72, 129]. Phenols (sometimes called phenolics), a class of compounds found in various essential oils, have been shown to exhibit inhibitory, microbicidal, and anti-biofilm properties against a wide spectrum of microbial species. This includes fungal genera (e.g., *Aspergillus, Fusarium, Penicillium, Trichophyton, Microsporum, Cryptococcus*), bacterial genera (e.g., *Bacillus, Staphylococcus, Streptococcus, Escherichia, Pseudomonas, Listeria, Enterobacter, Proteus),* and yeast genera (e.g., *Candida*) [135, 155, 160]. The antimicrobial mechanisms of phenols include: (1) alteration of membrane function and structure by interacting with membrane lipids and proteins disrupting their integrity and function, which leads to modifications in membrane potential and leakage of cellular content, (2) intracellular acidification and coagulation of cytoplasmic constituents, causing disturbances in cellular activities (e.g., ATP production); (3) inhibiting/inactivating important enzymes (e.g. DNA-gyrase) which causes disturbances in replication and transcription of DNA; (4) disturbing biofilm formation by affecting bacterial adherence mechanisms through the generation of ROS [117, 160]. Porphyrins exhibit antiviral and antimicrobial activity, primarily due to their high affinity to bind to cellular structures like membranes, proteins, and DNA, mixed with their ability to induce photosensitization and generate ROS [8, 143]. Carotenoids have been documented for their antimicrobial properties [102]. However, it is important to note that spiders lack the ability to synthesize carotenoids de novo [21], making them dependent on dietary sources to obtain these compounds. Lastly, xanthurenic acid, acquired from *T. clavata* golden dragline silk was shown to have a slight antibacterial effect against both Gram-negative (*Escherichia coli*) and Gram-positive (*Bacillus subtilis*) bacteria. This inhibitory effect was documented based on OD600 measurements in a LB broth medium tests [41]. No other studies showing antimicrobial activity for this compound could be found. However, xanthurenic acid has been documented to inhibit human glioma cells, as reported by Nayak and Buttar [112].

Taking all of that into account, most of the compound families present in silk pigments have demonstrated antimicrobial potential in other studies. The synthesis of these pigments likely involves a considerable metabolic cost, indicating that they serve crucial functions. Nonetheless, it is important to note that not all spider species produce pigments in their silk [32]. We propose that the protective role of cocoon pigments against insects may also involve an additional sensory ecology aspect. The color of the egg sac, which often appears conspicuous to the human eye, might instead blend with the background from the perspective of potential predators, thereby reducing predation pressure. Exploring these and other spectral properties of spider cocoons in order to understand how their parasitoids perceive them presents another intriguing research opportunity [17, 32] adding next bits of possible knowledge to the spider cocoon topic, which could be investigated in the research based on methods and ideas presented in Glenszczyk et al. [45].

Furthermore, Lammers et al. [77] documented the existence of agents with both insecticidal and antimicrobial properties in the volatilome (i.e., compound cloud) of the communal silk in social spider *Stegodyphus dumicola.* The authors noted that most of the identified substances were well-known in the literature, having been previously reported in the essential oils of plants, insects, and, notably, in microorganisms such as fungi, bacteria, and algae. This finding raises questions not only about the potential composition of compounds in spider cocoon silk but also about the structure of its potentially unexplored microbiome, which could be responsible for producing them.

## Ecological interactions

Life history theory provides an evolutionary framework for understanding how organisms allocate their limited resources across different stages of their life cycle, including growth, reproduction, and survival. The associated traits, such as brood size, offspring viability, reproductive effort, and parental investment, evolve under a constant natural selection to address specific ecological challenges determined by a changing environment [114, 142, 163]. Microorganisms are ubiquitous throughout the biosphere, influencing other organisms in a variety of ways, from imperceptible to beneficial, or even harmful. When these interactions are detrimental, they present a biotic ecological challenge that also drives the evolution of life history traits in other organisms to counteract or mitigate their effects [115, 124].

An animal’s ability to mount an adaptive and effective immune response against pathogenic microorganisms is known as immunocompetence [115]. This response usually comes with a range of trade-offs, as the available resources that can be allocated are often limited [80, 96, 107, 139, 157]. Measuring immunocompetence comes as problematic. For some groups of animals like spiders, the number of commercially available associated tests, assays, tools, etc., is very limited, and thus a scientist is constrained to products that are evolutionary conservative [33, 91]. Moreover, the assessments must always be done for a range of components, ideally within a single experiment [115], which not only increases the cost of such a venture but may also be difficult to achieve methodologically and requires good planning ahead. In some animals, the scope of immunological response for each of the developmental stages is largely unknown, which makes this issue even more difficult to navigate, but it does not mean that the scientists are not trying to understand it better [33]. It is important to acknowledge that while immunocompetence is connected to physiology and may be measured through tracking biological markers, it is also related to morphology and behavior in animals [139], which was proven in multiple studies [96, 133, 134, 157]. Last but not least, immunocompetence must be regarded from the perspective of a particular population and a habitat, rather than the whole species. Nevertheless, the ability to generate an immunological response by a given population also provides us with information about the capabilities of species as a whole during the evolutionary arms race [80].

The concept of immunocompetence is also relevant to spider cocoons, as they too are susceptible to environmental factors, which may disrupt the development and survival chances of juvenile spiders, thus negatively impacting the parental investment made by female spiders. Humphreys [60] discussed three levels of protection for the egg sac, which counter these environmental challenges. The first two—the dense webbing of the cocoon, and the granule coating of the eggs are hypothesized to serve both abiotic and biotic defense. In addition, they are supported by the third, behavioral level, which is maternal guarding of the egg sac. These levels seem to correspond with the outlined morphological, physiological, and behavioral domains of immunocompetence, and all of them seem to show a great variance across different species of spiders.

### Microbial dynamics

Spider cocoons are formed in environments and habitats that are far from sterile, which could explain the evolutionary necessity of equipping them with antimicrobial properties. Furthermore, different spider species occupy distinct ecological niches, which may influence the level of exposure their cocoons may have to microorganisms. For instance, the model genera *Parasteatoda* sp. (Theridiidae) and *Pardosa* sp. (Lycosidae) exhibit different strategies in cocoon management, shaped by their respective habitats. *Pardosa* sp. (ground-dwellers) spiders transport their cocoons across the substrate, whereas *Parasteatoda* sp (web-builders) suspend their cocoons away from the ground on pre-spun webs. Given that the substrate is a known reservoir of pathogenic microorganisms [78], one could hypothesize that *Pardosa sp*. cocoons, due to their more frequent contact with the substrate and the associated increased risks of mechanical damage and pathogenic exposure, might exhibit higher immunocompetence compared to those of *Parasteatoda sp.*, whose cocoons, suspended on webs, face different ecological challenges. Another important matter concerns the microbial communities associated with egg sacs. Are there notable differences between individual spiders of the same species, or is the composition of microorganisms relatively consistent within a species, reflecting a unique fingerprint? Additionally, how much do microbial communities vary between different spider species, and are there taxa that are consistently present on the egg sacs of every spider species?

Currently, there is compelling evidence that spider silk is not sterile and can host microorganisms [40]. Liu [86] conducted a taxonomic analysis of possible bacteria on egg sac silk of *Lactrodectus hesperus* using next-generation sequencing (NGS), and found the presence of genera such as: *Bacillus, Delftia, Enterococcus, Erysipelotrichaceae, Gilliamella, Lactobacillus, Lactococcus, Mycobacterium, Pelomonas, Pseudomonas, Sporosarcina, Staphylococcu*s. The study further highlights the capacity of bacteria to adhere to silk fiber surfaces and establish biofilm formation within a few hours. Notably, the study also reveals a significant inhibition of *Bacillus megaterium* growth by egg sac silk in microbial culturing experiments. This finding aligns with observations by Wright and Goodacre [165], who reported that silk from *Tegenaria domestica* (Agelenidae) inhibited *Bacillus subtilis* growth, and suggested the presence of antibacterial compounds on spider silks. Recently, Tsiareshyna et al. [152] reported the presence of other bacteria, namely *Novosphingobium* sp. and *Microbacterium* sp., on *Trichonephila clavata* (Nephilidae) spider webs. These bacteria are believed to enhance silk properties by depositing bacterial EPS (exopolysaccharides) onto the silk’s surface. Both species have been associated with soil, and previous studies have demonstrated that *Microbacterium* sp. exhibits antifungal activity [166]. Other known interactions between microorganisms and spider silk have been documented in the social spider *Mallos gregalis*, where yeast colonies residing on the silk produce a sweet odor that attracts additional prey. Microorganisms associated with the silk are thought to arise from prey remnants incorporated into the silk matrix, with partially consumed prey serving as a nutrient-rich medium for microbial growth. This phenomenon underscores a unique and intricate interspecific relationship. Notably, researchers have observed that the sweet odor transitions to an ammonia-like scent prior to spider colony collapse or mass emigration, suggesting that the silk-associated microbiome represents a dynamic and temporally variable system [146].

Conversely, several studies [1, 28, 53] have reported fungal infestations of spider cocoons. Horel and Gunermann [1] have observed that the presence of female spider prevented cocoon’s molding, but were uncertain towards the protection mechanism that ensured this. They hypothesized that it could be via additional weave of silk fibres [121] or “some antiseptic fluid” injected through the egg sac’s envelopes [30]. Mongkolsamrit et al. [103–106] identified fungi genera such as *Gibellula*, *Hevansia*, *Jenniferia*, *Polystromomyces*, and *Bhushaniella*, which display morphological adaptations enabling them to infiltrate egg sacs. For documented instances, there was a visible growth of mycelium covering the cocoon, which seemed to be a clear indicator showcasing a reproductive failure, as the eggs from such a cocoon were unlikely to develop. These fungi species could be predominantly found in leaf litter, on the ground, beneath leaves, or on plant stems, meaning their occurrence was rather abundant in spiders’ habitat. Notably, the authors emphasized that fungal attacks on cocoons are relatively uncommon, with both juvenile and adult spiders being particularly vulnerable to fungal parasitism. Nyffeler and Hywel-Jones [116] have documented ongoing discoveries and descriptions of new species of spider-pathogenic fungi. Despite difficulties in estimating their true diversity, it was argued that the number of yet undescribed species could easily exceed one thousand. This observation, however, raises intriguing questions about the notable absence of comprehensive reports of mutualistic relationships between spiders and fungi. Does this suggest that, in most cases, fungi predominantly form parasitic or opportunistic associations with spiders? In other invertebrates, such as insects, fungi are known to form mutualisms in which antimicrobial protection is a widespread phenomenon, especially if one of developmental stages include frequent contact with soil or plants [20]. In addition, authors point out that the mutualistic systems often overlap, arguing that fungi species which participate in protective mutualism providing antimicrobial defense often also partake in nutritional mutualism providing sustenance.

Other recent studies [109] have documented the presence of maternally inherited endosymbiotic bacterial species, including *Rhabdochlamydia*, *Wolbachia*, and *Spiroplasma*, in the eggs of the brown widow spider, *Latrodectus geometricus* (Theridiidae). Additional endosymbionts, such as *Rickettsia* and *Cardinium*, have also been reported in other spiders [154]. Endosymbionts are known to impact their hosts in various ways, including modulating sexual reproduction, influencing nutritional processes, or enhancing resistance to pathogens [51, 162]. Masson and Lemaitre [93] have shown that in most cases they remain difficult to culture via traditional methods, and in invertebrates may be transmitted both horizontally and vertically. The vertical transmission includes deposition through transovarial transfer, milk glands, egg smearing, egg capsules, egg jelly, and brood smearing. The ways of transmission of these endosymbionts open another question regarding spider eggs, which remains in a close proximity to the considerations we have provided in Eggs and Their Protection. Could the Humphreys [60] second level of protection be further enhanced by potential endosymbiotic deposits? Mowery et al. [109] propose that some endosymbiont strains may confer benefits to their spider hosts. This hypothesis is supported by the observations of high vertical transmission and significant prevalence rates, which speak against strong pathogenic roles in spider populations. Studies in other arthropods suggest that certain endosymbionts, such as *Wolbachia*, may enhance immune priming, meaning that the presence of this endosymbiotic bacteria elevates the basal immune status of the infected host, which results with strong responses whenever the host is subsequently infected with other pathogens [147].

The microbial dynamics associated with spider egg sacs appear to be strongly influenced by environmental factors, although the precise role and nature of these interactions remain poorly understood. Efforts have been made to elucidate some of the potential functional contributions via comparative genomics, particularly in relation to endosymbionts, which have been implicated in the biosynthesis of essential amino acids and vitamins [51]. These findings suggest that endosymbionts can play multifaceted roles in supporting spider physiology and ecology. However, considering other types of microorganisms, the vast diversity of spider species and the range of habitats they inhabit, this area of research still represents a relatively small component of the broader picture, as the potential ecological relationships that microorganisms could form with spider webs, and most importantly—cocoon silk and cocoon eggs—remain insufficiently characterized. More research employing biochemical, metabolic and metagenomic analyses and related methods could help to know better about the microbiome of spider cocoons.

This approach could help to address previously mentioned key questions, such as the structure and possible variations in microbial communities between individuals, populations, and species, and whether certain microorganisms are consistently associated with spider cocoons. On top of that, additional studies of the microbial communities [63] from the natural spider habitats could further offer valuable insights into whether certain microorganisms can transfer from the habitat onto the cocoon. The research in this area could also uncover uncultivable microorganisms and determine whether other symbionts capable of producing antimicrobial or other compounds are present, as well as identify potentially unknown or pathobiontic species.

### Environmental dynamics

The presumed antimicrobial properties of spider cocoons represent a significant and underexplored immunological aspect, yet equally intriguing are the spider habitats themselves, which often highlight the existence of a broader scale of ecological strategies, which come into play regarding spider cocoon protection. Each habitat inherently imposes interactions between spiders and microorganisms, as microorganisms are ubiquitous across all environments. The composition and characteristics of the microbiome may vary significantly depending on environmental conditions resulting in the emergence of specialized groups adapted to distinct ecological niches. There are no known environments inhabited by spiders, which would lack interactions with microorganisms. In response to these persistent pressures, spiders have evolved a range of defense strategies, many of which remain to be described. Among these is the egg sac, notable for its unique protective properties. There are, however, other evolutionary defense mechanisms that have emerged, some of which involve the incorporation of environmental elements, which were noted to enhance microbial resistance. These revelations also can pave the way for the discovery of novel antimicrobial compounds.

**Fig. 4:**
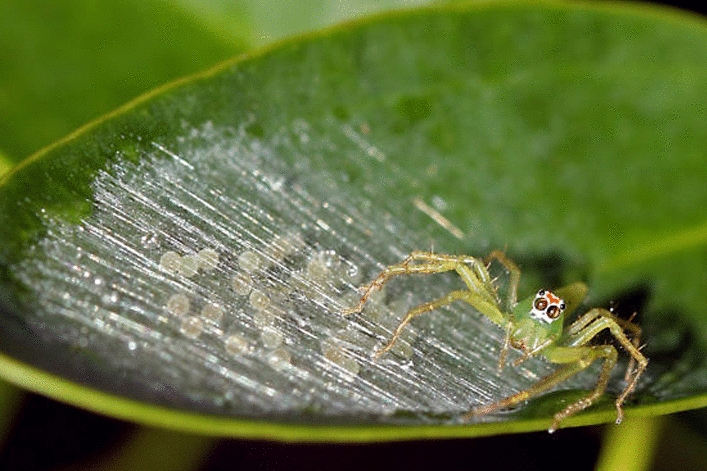
*Lyssomanes viridis* with her egg sac. Photography by Amanda Carta (@MnMCarta). Used with the author’s permission

In a study by Tedore and Johnsen [144], the plant-dwelling spider *Lyssomanes viridis* (Salticidae) was observed to preferentially construct its egg sacs on the leaves of the *Liquidambar styraciflua* tree (commonly known as Sweetgum), a species known for producing a volatilome rich in broad-spectrum antimicrobial compounds. The researchers noted that *L. viridis* constructs lightly woven egg sacs with eggs spaced relatively far apart, potentially increasing eggs exposure to antimicrobial volatiles emitted by the leaves (Fig. 4). The study demonstrated that hatching success was significantly reduced when egg sacs were built on other sympatric plant species or on plastic controls, suggesting a potential immuno-ecological adaptation in which the antimicrobial properties of *L. styraciflua* enhance reproductive success in a spider species.

There are species of spiders that engage into mutualistic symbiosis with carnivorous pitcher plants (Nepenthaeae), (e.g., *Misumenops nepenthicola*, *Synema obscuripes*, Thomsidae). *Misunemops nepenthicola* exhibits a marked preference for *Nepenthes gracialis* over *Nepenthes rafflesiana,* while *Synema obscuripes* forms a relationship with *Nepenthes madagascarensis*. Both spider species attach their cocoons to the inner side wall of a pitcher, and are noted to aggressively guard their cocoons [64, 75, 126]. Digestive fluid of pitcher plants serves as a reservoir for diverse bacterial communities spanning multiple phyla [27]. Observations by Karl and Bauer [64] reveal that female spiders frequently come into direct contact with both the pitcher’s digestive fluid and the carcass. These interactions present intriguing avenues for exploration, particularly regarding the complex interrelationships between the cocoon, the female spider, and the microbiota within this ecological niche, as well as the adaptive traits of the cocoons and the underlying factors influencing plant selection preferences in these spiders.

**Fig. 5:**
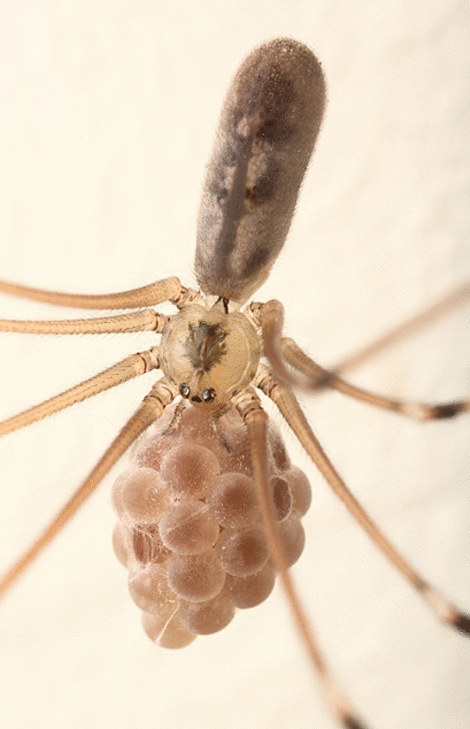
*Pholcus phalangioides* female with egg sac. Photography by Adam Poledníček. Used with the author’s permission. Other photographic documentation of pholcid egg sacs available at: (http://www.pholcidae.de/)

There are genera that produce poor or even absent silk cocoons, as observed in the Pholcidae family (Fig. 5), where the egg sacs are usually wrapped in only a few thin threads of silk. These cocoons are consistently carried by the chelicerae, with a slightly denser, circular mat of silk formed at the point where the egg sac is held [58]—Fig. 1). Hajer et al. [50] pointed out that silk does not play any true protective role in those spiders, and it is rather the strong thick chorion, which protects developing embryos. The scientists also observed that the low diversity and number of the spinning glands in a given spider species may be connected to the structure of its cocoon.

Huber and Eberle [58], beyond documenting pholcid egg sacs, have uncovered key morphogenetic patterns, including a relationship between egg number, egg size, and female body size, with smaller pholcid species tending to produce larger eggs. They also identified a subtle correlation between environment and egg characteristics, noting that ground-dwelling species, relative to their body size, had smaller eggs than leaf-dwelling species. Other than that, their findings suggest that clutch size is influenced by both body shape and microhabitat, further advancing the knowledge of pholcid reproductive strategies. Authors have shown that body size, body shape (particularly the length of the abdomen), and preferred microhabitat can interact in complex ways. While their analyses provide additional evidence of a multifaceted network of variables and relationships in life history traits, the defense mechanisms of these egg sacs still remain a great unknown. Given the substantial energetic costs associated with the production of defense mechanisms [10], we could suppose that the pholcid egg sacs may result from a trade-off among three levels of cocoon protection described by Humphreys [60], in which biotic and behavioral strategies are prioritized over abiotic protection in order to maximize survival of the offspring. On the other hand, there are other possible explanations including differences in energy allocations for growth and development, which obviously cannot be ruled out.

## Conclusion

The presumed antimicrobial properties of spider cocoons remain a debatable scientific issue, which we refer to in the title as an ‘apple of discord’. It means that for now this topic lacks a definitive answer, as research in this field frequently yields conflicting results. Numerous questions remain unresolved, overlooked, or unexplored, highlighting the significant potential for this area to generate new knowledge.

Given the vast diversity of spiders, the answer to this question likely varies depending on ecological, evolutionary, and environmental factors. For instance, some species may not exhibit antimicrobial properties in their cocoons due to mutualistic relationships that negate the need for additional resource investment in such defenses. Others might not require these antimicrobial adaptations due to the abiotic protective qualities of silk itself and the absence of specialized threats (e.g., fungal species) able to target and penetrate the cocoons. Conversely, certain spider species may indeed demonstrate antimicrobial properties, driven by ecological pressures that necessitate equipping cocoons with such defensive mechanisms. Even further complicating the exploration of this topic are methodological challenges, which must be addressed to achieve more clearer insights.

We hope that our efforts to consolidate existing knowledge and provide a comprehensive overview of this topic, combined with our more ecological perspective, will contribute to advancing research and investigations in this field. At the same time, we acknowledge that, despite our best efforts to address the most critical aspects, certain dimensions of this subject may not have been fully captured. We view this as an encouraging sign, as it opens additional opportunities for dialogue, inspiration, and, most importantly, the further findings in the domains of this research.

## Data Availability

Not applicable.
